# Caco2/HT-29 In Vitro Cell Co-Culture: Barrier Integrity, Permeability, and Tight Junctions’ Composition During Progressive Passages of Parental Cells

**DOI:** 10.3390/biology14030267

**Published:** 2025-03-06

**Authors:** Elena Donetti, Paola Bendinelli, Margherita Correnti, Elena Gammella, Stefania Recalcati, Anita Ferraretto

**Affiliations:** Department of Biomedical Sciences for Health, University of Milan, 20133 Milan, Italy; paola.bendinelli@unimi.it (P.B.); margherita.correnti@unimi.it (M.C.); elena.gammella@unimi.it (E.G.); stefania.recalcati@unimi.it (S.R.); anita.ferraretto@unimi.it (A.F.)

**Keywords:** intestinal epithelial barrier, transepithelial electrical resistance, claudin 2, occludin, MUC-2, co-culture, western blot, transmission electron microscopy, immunofluorescence

## Abstract

Epithelial linings are crucial for the maintenance of physiological barriers. The intestinal epithelial barrier (IEB) consists of enterocytes through tight junctions and mucus-secreting cells and can undergo physiological modifications throughout life. To reproduce as closely as possible the IEB main features over time, in vitro co-cultures of Caco2/HT-29 70/30 formed by parental Caco2 and HT-29 cells sub-cultivated for more than 40 passages were set up. The measurements of the transepithelial electrical resistance (TEER) identified two populations: physiological TEER co-cultures (PC) with values > 50 Ωcm^2^ formed by parental cells with fewer than 40 passages, and leaky TEER co-cultures (LC) with values < 50 Ωcm^2^ formed by parental cells with more than 40 passages. In LC, paracellular permeability increased in parallel. By immunofluorescence and Western blot analysis, an increase in claudin 2 was observed in LC vs. PC, with no differences in occludin expression. MUC-2 immunoreactivity was stronger in PC than in LC. LC also showed an enhanced vulnerability to TNFα+IFN-γ. These results reproduce the main morpho-functional modifications reported in the human leaky/aged gut and support the usefulness of our in vitro cell model for studying the molecular processes underlying these modifications and testing drug/nutraceutical treatments to ameliorate leaky gut aging.

## Simple Summary

In our body, the lungs, skin, and gut represent anatomical barriers that face different environmental stimuli. These structures can undergo modifications under specific conditions like illness, dysbiosis, apoptosis, and aging. In dysbiosis, the loss of commensals and colonization of opportunistic pathogens modify TJ permeability. Apoptosis causes uncontrolled TJ permeability. The need to set up experimental models mimicking these changes is an actual challenge. In the gut, absorptive cells, i.e., enterocytes, and mucus-secreting cells, i.e., goblet cells, contribute to the intestinal barrier. We characterized the modifications occurring in an in vitro co-culture of Caco2 (enterocytes)/HT-29 (goblet cells) cells over time. Co-culture formed by parental Caco2 and HT-29 cells cultivated at high passages showed increased permeability compared to co-culture formed by parental cells at low culture passages. These modifications were linked to a switch in the protein composition of the junctions involved in the maintenance of the barrier. Mucus was also reduced in co-culture from high-passage parental cells. The features herein reported indicate an impairment of the intestinal barrier comparable to that found in the aged gut. This in vitro experimental model offers the possibility of investigating the impact of nutrients, drugs, and supplements on the intestinal epithelial barrier over time.

## 1. Introduction

The maintenance of physiological body barriers such as the lungs, skin, and gut is crucial for the protection of the internal organs against a huge variety of exogenous stimuli [1]. A key role in the formation of such anatomical structures is played by the epithelial linings, each of them with morpho-functional, intrinsic, and distinctive properties depending on the body district. In the intestinal epithelial barrier (IEB), enterocytes and goblet cells actively participate in the functions of the barrier thanks to the formation of tight junctions (TJs) and mucus synthesis and secretion [2]. TJs are dynamic structures located on the apical side of the enterocytes, in which the outer leaflet of the epithelial cytoplasmic membranes between adjacent cells seems to fuse, creating “kissing points”. This efficient organization regulates paracellular permeability by controlling the size of the intercellular space, considering that cell permeability is strictly related on one side to TJ architecture and on the other side to the dimension of the molecules. A complex network operates in TJs and consists of integral and peripheral proteins [3]. The former group, characterized by four-pass transmembrane domains, includes claudins, comprising at least 27 members, occludin, tricellulin/MarvelD2, MarvelD3, and junctional adhesion molecules (JAMs) [4,5]. These proteins constitute the skeleton of the TJs. Peripheral scaffold proteins, such as zonula occludens (ZOs) 1, 2, and 3, connect the cytoplasmic tail of the transmembrane proteins to the actin cytoskeletal filaments [6]. Different claudin subtypes can be co-expressed in the same cells and differently expressed in each epithelium, thus determining the physiologically specific barrier properties [7]. 

Mucin 2 (MUC-2), constituting the main component of the loose mucus layer, is secreted by goblet cells in the crypts and along the villi [8], cooperates in IEB maintenance, and seems to be essential for disease prevention [9]. The mucus forms a continuous layer covering the epithelial surface and represents an important chemical barrier against endogenous and exogenous irritants with a relevant role in nutrient absorption [10].

The increased permeability of IEB is a typical feature of the leaky gut [11], a condition that allows the passage of substances, such as toxins, microorganisms, fragments, and waste material produced by digestion, from the gastric lumen to the bloodstream. The increased permeability is associated with the localization, quantity, and function of IEB, which comprises, beyond TJs, also outer components, such as commensal microbiota [11,12]. These events lead to inflammation and excessive reactive oxygen species (ROS) production, all risk factors for the onset of different chronic and aging-related diseases [13]. The leaky gut theory supports the onset of chronic inflammatory bowel diseases, currently known as IBDs, and irritable bowel syndrome (IBS); indeed, it has been observed that patients suffering from these disorders have higher intestinal permeability than healthy subjects [14]. Besides gut health problems, a connection seems to exist between increased IEB permeability and autoimmune diseases, allergies, food sensitivities, diabetes mellitus, and pathologies of the central nervous system [11]. In this scenario, the process of gut aging presents some features and events very similar to those of leaky gut, for instance, major IEB permeability due to an upregulation of claudin 2, as observed in animal models of colitis and IBD patients, low-grade chronic inflammation known as inflammaging, and a change in the intestinal microbiota [15].

The knowledge of the multiple and uncontrolled factors involved in intestinal permeability modification driving a leaky gut development represents a significant challenge that requires valuable experimental in vitro cell culture models, allowing us to study at the molecular level all involved mechanisms, and at the same time to overcome evident ethical limits of animal experimentation with substantial savings in terms of time and cost [16].

More than one cellular intestinal model has been studied over the years, but Caco2 cells, derived from a human colon adenocarcinoma, still represent the most used one, although they present several characteristics different from the human small intestine, such as the absence of mucus and a restricted permeability, which is more compatible with the colon region [17,18]. To improve the Caco2 intestinal model with the presence of mucus-secreting cells, we standardized an in vitro co-culture consisting of Caco2 and HT-29 cells in a ratio of 70/30 (70/30 Caco2/HT-29), starting from parental cell lines previously differentiated from 20 to 40 passages in culture to minimize the heterogeneity of the cells, obtain the maximum reproducibility possible, and standardize the methodology [19]. This co-culture was also characterized by transepithelial electrical resistance (TEER), an index of the resistance to the flux of substances such as ions and electrolytes through the paracellular way, like that of the small human intestine [17]. Successively, the 70/30 Caco2/HT-29 cell co-culture has demonstrated decreased TEER values and inhomogeneous distribution of claudin 1 associated with increasing passage numbers of the parental Caco2 and HT-29 cells [20], thus suggesting that our model reproduces some pivotal features of leaky gut.

The present study aimed to complete the characterization of the morphological and functional modifications in Caco2/HT-29 cell co-culture over time. First, we studied TEER and paracellular permeability as the number of passages of parental cells increases. Secondly, considering that (i) epithelial permeability is strictly related not only to barrier-forming proteins as claudin 1 but also to pore-forming proteins as claudin 2 [21], and (ii) occludin contributes to the TJ-related barrier [22], we analyzed by indirect immunofluorescence the distributions of claudin 2 and occludin. In parallel, Western blot analysis was performed on the same samples. To complete the tableau of IEB features, MUC-2 immunofluorescence analysis was then carried out to define the intracellular localization of the mucus, also verified by transmission electron microscopy. On the basis of the TEER measurements, we observed that, as the number of passages of parental cells increased, a significant reduction in TEER associated with increased permeability occurred, accompanied by an increase in claudin 2 but not of occludin, and a decrease in MUC-2, all features attributable to a leaky gut, especially during aging and/or IBD onset.

## 2. Materials and Methods

Unless otherwise specified, all cell culture media and reagents were from Merck Italy (Milan, Italy); FBS was from EuroClone Ltd. (West Yorkshire, UK); Tumor Necrosis Factor-α (TNF-α) and Interferon-γ (IFN-γ) were from Peprotech (Cranbury, NJ, USA).

### 2.1. Cell Cultures

The cell lines HT-29 (BS TCL 132) and Caco2 (BS TCL 87), both from human colon carcinoma, were purchased from Istituto Zooprofilattico Sperimentale (Brescia, Italy). Cells were cultured in Roswell Park Memorial Institute (RPMI) 1640 containing 13.9 mM glucose supplemented with 10% Fetal Bovine Serum (FBS), 2 mM l-Glutamine, 0.1 mg/L streptomycin, 100,000 U/L penicillin, and 0.25 mg/L amphotericin B in a 37 °C humidified atmosphere with 5% CO_2_. Cells were sub-cultivated at post-confluence for Caco2 [23] and at 90–100% confluence for HT-29 [24]. Co-culture of Caco2 and HT-29 was assessed by seeding a mixture of differentiated Caco2 and HT-29 cells in a 70/30 ratio to obtain a mixed population of enterocytes and mucus-secreting cells similar as much as possible to the human small intestine, as previously described [19], from here on indicated as co-culture. To set up the co-culture, Caco2 cells were used from the 20th to the 55th passages and HT-29 cells from the 8th to the 42nd passages. The co-cultures were plated with a 40,000 cells/cm^2^ density and maintained for 10 days (6 days after complete confluence) before all the experiments.

### 2.2. Functional Properties of IEB: Transepithelial Electrical Resistance (TEER) Measurement and Intestinal Paracellular Permeability Assay

Co-culture was grown in a 24-Transwell plate (Transwell Millicell, Cell Culture Insert PET 1 μm, Millicell 24-Well Receiver Tray, Merck KGaA, Darmstadt, Germany). TEER was measured with a Millicell ERS system (Millipore Corporation, Burlington, MA, USA) at 37 °C 24 h after changing the growth medium. TEER values obtained in the absence of cells (blank) were subtracted from all sample values. For each condition, at least three wells were set up, and TEER values were measured at three points of each well for all experiments. Results were expressed as Ωcm^2^ [19].

The fluorescent probe Lucifer Yellow (LY) was used to measure the paracellular permeability [19]. After the TEER measure, a solution of 100 μM LY in HBSS/5mM HEPES was added to the apical chamber of each well. After two hours, both apical and basolateral solutions were collected, and 100 µL from each solution was used to measure the fluorescence intensity at an excitation wavelength of 475 nm and emission wavelength of 540 nm, using GloMax Discover (Promega Corporation, Madison, WI, USA). A standard curve allowed us to convert the fluorescence values into concentration values. To study the functional properties of IEB after the application of an inflammatory stimulus, co-culture was seeded in a transwell and was treated with or without a mixture (MIX) of TNF-α (50 ng/mL) and IFN-γ (50 ng/mL) prepared in the growth medium [25,26,27]. The MIX was administered in the basolateral side, and after 48 h, TEER and paracellular permeability were measured. The experiments were repeated three times.

### 2.3. Morphological Properties of IEB: Immunofluorescence and Transmission Electron Microscopy Analysis

Co-culture was seeded and cultured on glass coverslips, and cells were fixed with 75% ethanol in PBS 0.1 M pH 7.4 at −20 °C for 30 min, washed with PBS 0.1 M pH 7.4 (3 × 5 min), and permeated with 75% acetone in PBS 0.1 M pH 7.4 at −20 °C for 3 min. Table 1 summarizes the antibodies and protocols for immunofluorescence.

After three washings, nuclei were counterstained and mounted with Fluoroshield^TM^ (Merck Italy, Milan, Italy) with DAPI [19]. For each antibody, a negative control was performed by omitting the primary antibody and replacing it with PBS 0.1 M pH 7.4. A confocal Nikon A1/A1R (Nikon, Tokyo, Japan) was used to collect data using NIS Elements F4.60.00 software version AR 5.42.06. At least four fields for each sample and each original magnification (40×, 60×, and 100×) were analyzed.

For the nuclear size, we measured the dimension of 10 nuclei/microphotograph in three microphotographs for each group (a total of 30 measurements for each group) with the NDP.view2 Image viewing software version U12388-01 (Hamamatsu Photonics K.K., Hamamatsu City, Shizuoka, Japan).

For transmission electron microscopy (TEM) analysis, cells were seeded in tissue culture dishes and fixed for 60 min at room temperature with 3% glutaraldehyde buffered in 0.1 M cacodylate buffer (pH 7.4). After three washings of 10 min at room temperature with the same buffer, samples were post-fixed in 1% osmium tetraoxide diluted in 0.1 M cacodylate buffer at room temperature for 30 min and in uranyl acetate 2% in distilled water for 15 min at room temperature. Then, samples were progressively dehydrated in 30%, 50%, 70%, 80%, 90%, and 100% ethanol, then embedded in Epon/Araldite 1:3 for 48 h at 60 °C, as previously described [19]. Ultrathin sections (75–80 nm thick) were obtained with Cryo-Ultramicrotome RMC PowerTome XL (Leica, Wien, Austria), stained with uranyl acetate and lead citrate, and observed with Talos 120 (Thermo Fisher Scientific, Waltham, MA, USA).

### 2.4. Western Blot Analysis

Cells were lysed at 4 °C with RIPA buffer and centrifuged for 30 min at 14,000× *g* at 4 °C. The supernatant was ultracentrifugated at 30,000× *g* at 4 °C and then used for Western blot experiments. Antibodies were used for Western blot analysis according to the manufacturer’s recommendations. Immunoblots were incubated overnight at 4 °C with the following primary antibodies: mouse monoclonal anti-claudin 2 (Invitrogen, Monza (MB), Italy, #32-5600, 1:500) and rabbit polyclonal anti-occludin (Invitrogen, Monza (MB), Italy, #40-4700, 1:500). Immunoblots were then incubated with secondary antibody α-rabbit/mouse (Cell Signaling Technology, Euroclone, Milan, Italy, #7074 and #7076, 1:1000) for 1 h at RT. Mouse monoclonal anti-HSP60 antibody (Invitrogen, Monza (MB), Italy, #MA3-012) and rabbit monoclonal anti-β-actin antibody (Cell Signaling Technology, Euroclone, Milan, Italy, #8457) were used as internal controls (1:1000). Quantification of the signal was obtained by chemiluminescence detection using the ECL method (Amersham, Pero, Italy) on the ChemiDoc™ Touch Imaging System (Bio-Rad, Milan, Italy) and analyzed with ImageLab 5.2.1 software with the values being calculated after normalization to the amount of HSP60 or β-actin.

### 2.5. Statistical Analysis

All data are reported as means ± standard deviation (SD) and derived from at least three independent experiments. Each experiment consisted of at least n = 3 replicates for each sample. Statistical significance was determined using one-way or two-way ANOVA followed by Tukey’s multiple comparisons test, and Student’s *t*-test for nuclei dimension comparison (GraphPad Prism, version 10.1.0, GraphPad Software, San Diego, CA, USA). For the Western blot analysis, statistical significance was assessed using Welch’s *t*-test (GraphPad Prism). *p*-values < 0.05 were considered statistically significant [28].

## 3. Results

### 3.1. Functional Properties of Caco2/HT-29 70/30 Co-Culture in Basal Condition and After Pro-Inflammation Cytokine Treatment with the Increasing Passage Number of the Parental Cells

**Figure 1:**
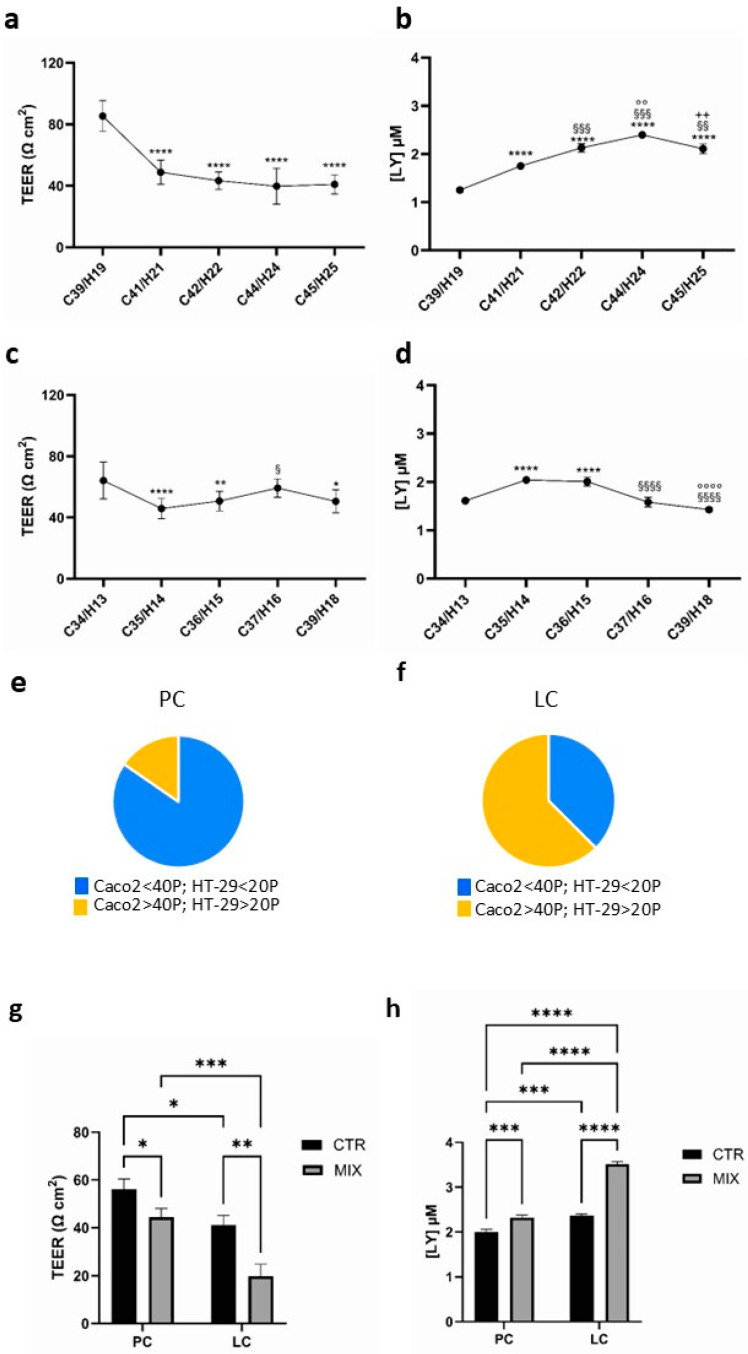
Functional properties of Caco2/HT-29 70/30 co-culture in basal condition and after pro-inflammation cytokine treatment with the increasing passage number of the parental cells. (**a**,**c**) TEER values, expressed as Ωcm^2^. Each point represents the mean ± SD. (**b**,**d**) Paracellular permeability to LY, expressed as [LY] in the basolateral chamber of the transwells. Each point represents the mean ± SD. In (**a**,**b**) **** *p*-value < 0.0001 vs. C39/H19; §§ *p*-value < 0.01, §§§ *p*-value < 0.001 vs. C41/H21; °° *p*-value < 0.01 vs. C42/H22; ++ *p*-value < 0.01 vs. C44/H24. In (**c**,**d**) * *p*-value < 0.05, ** *p*-value < 0.01, **** *p*-value < 0.0001 vs. C34/H13; § *p*-value < 0.05, §§§§ *p*-value < 0.0001 vs. C35/H14; °°°° *p*-vale < 0.0001 vs. C39/H18. In (**a**–**d**) Cxx/Hyy, co-culture derived from Caco2 at the xx passage of sub-cultivation and HT-29 at the yy passage of sub-cultivation. (**e**,**f**) Graphical representation of the percentage distribution of PC and LC obtained from the parental cells with different numbers of sub-cultivations. (**g**) TEER values, expressed as Ωcm^2^. Each bar represents the mean ± SD. (**h**) Paracellular permeability to LY expressed as [LY] in the basolateral chamber of the transwells. Each bar represents the mean ± SD. In (**g**,**h**), CTR: control; MIX: 50 ng/mL TNF-α plus 50 ng/mL IFN-γ; * *p*-values < 0.05, ** *p*-value < 0.01, *** *p*-value < 0.001, **** *p*-value < 0.0001; in (**e**–**h**), PC: physiological co-cultures with TEER > 50 Ωcm^2^, in (**g**,**h**) Caco2 from the 34th to 37th passages and HT-29 from the 13th to 16th passages; LC: leaky co-cultures with TEER < 50 Ωcm^2^, in (**g**,**h**) Caco2 from the 35th to 44th passages and HT-29 from the 14th to 25th passages.

The functional characteristics of the co-cultures were studied in relation to the number of sub-cultivations of parental cells. Figure 1a,c show the TEER trend in co-cultures set up with parental cells as their number of passages in culture increased and represent two of the seven experiments performed. In Figure 1a, the co-culture formed with Caco-2 at the 39th passage and HT-29 at the 19th passage (C39/H19) shows a value of TEER of 85.38 ± 10.06 Ωcm^2^, which significantly decreased to 48.88 ± 7.82 Ωcm^2^ till 39.74 ± 11.56 Ωcm^2^ as the number of passages of parental cells increased (*p*-value < 0.0001). The course of paracellular permeability (Figure 1b) was consistent with TEER values, progressively and significantly increasing as the sub-cultivation of parental cells augmented (from 1.4- to 1.92-fold vs. the starting point C39/H19, *p*-value < 0.0001). In Figure 1c, the TEER value at the starting point of C34/H13 co-culture was 64.17 ± 12.07 Ωcm^2^ and significantly decreased following the progressive two sub-cultivations of parental cells (0.71- and 0.78-fold vs. the starting point C34/13 (*p*-value < 0.0001 and *p*-value < 0.01, respectively). In this co-culture, the TEER values ranged between 64.17 ± 12.07 Ωcm^2^ and 50.52 ± 7.56 Ωcm^2^. Similarly, the paracellular permeability reflected the trend of TEER, increasing by about 26% versus the starting point (C34/13) after two passages in culture (Figure 1d).

A TEER value ranging between 50 and 100 Ωcm^2^ corresponds to a healthy in vivo human small intestinal epithelium [17]; thus, values below 50 Ωcm^2^ indicate an IEB more permeable than the physiological condition and are considered a leaky gut. On this basis, we identified two populations of co-cultures, i.e., physiological TEER co-culture (PC), with values > 50 Ωcm^2^, and leaky TEER co-culture (LC), with values < 50 Ωcm^2^. Of note, among 13 PC, with TEER values ranging between 53.83 ± 9.11 and 85.38 ± 10.66 Ωcm^2^, 85% of co-cultures were set up with Caco2 having a passage number lower than 40 and HT-29 lower than 20. Among 16 LC, with values ranging between 33.80 ± 8.81 and 49.49 ± 7.76 Ωcm^2^, 63% were set up with Caco2 having a passage number higher than 40 and HT-29 higher than 20 (Figure 1e,f).

Since a leaky gut is characterized by a different response to inflammatory stimuli [14], we incubated the co-cultures with a mixture of TNF-α and IFN-γ (MIX) and measured TEER and paracellular permeability. The exposure to the cytokines caused a significant reduction in TEER associated with increased permeability in both populations, attesting to the occurrence of an inflammatory condition (Figure 1g,h). Interestingly, considering TEER values, LC appeared more susceptible to the inflammatory stimulus than PC: after cytokine treatment, TEER dropped to 44.40 ± 3.70 Ωcm^2^ (75.06 ± 8.6% vs. CTR) in PC, and to 19.88 ± 4.97 Ωcm^2^ (47.29 ± 13.5% vs. CTR) in LC, indicative of a significantly higher reduction in LC (*p*-value < 0.05). A parallel trend in paracellular permeability was detected in LC vs. PC (*p*-value < 0.05).

### 3.2. Molecular Composition of IEB: Tight Junctions and MUC-2 Analysis

**Figure 2:**
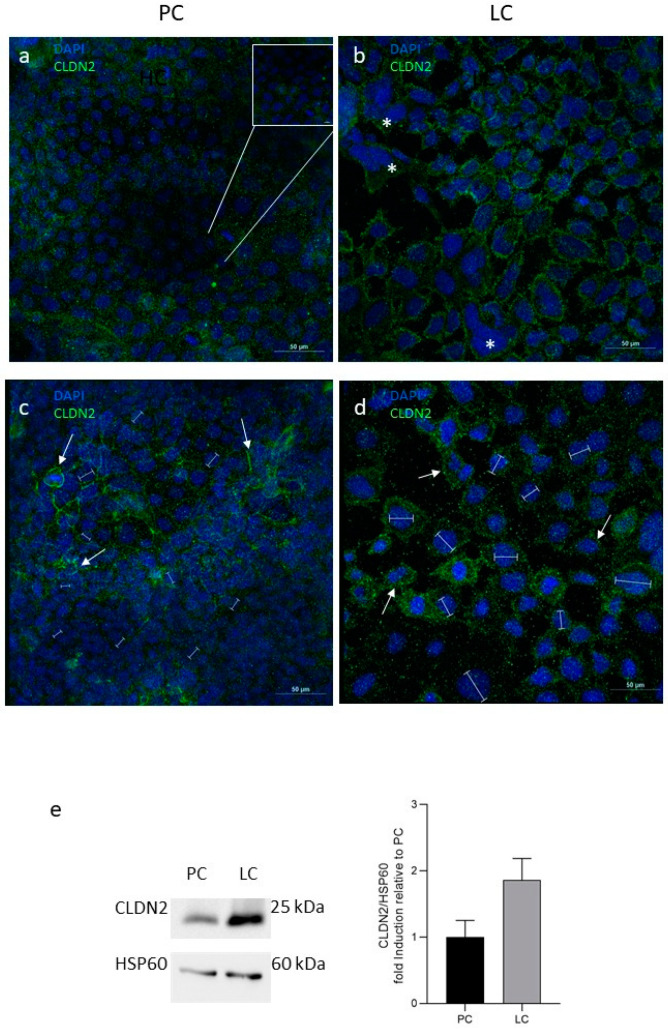
Analysis of the molecular composition of the tight junctions in Caco2/HT-29 70/30 co-culture showing different TEER values. (**a**,**b**) Indirect immunofluorescence for claudin 2; (**c**,**d**) immunofluorescence for occludin. (**e**) Western blot analysis for claudin 2. PC: physiological co-cultures with TEER > 50 Ωcm^2^, Caco2 30th passage and HT-29 21st passage; LC: leaky co-cultures with TEER < 50 Ωcm^2^, Caco2 54th passage and HT-29 41st passage. Nuclei are counterstained with DAPI. Asterisks in (**b**) indicate multinucleated cells. White arrows in (**c**,**d**) indicate the different occludin staining, respectively, in PC and LC. Labels in (**c**,**d**) indicate the nuclear dimension. Bars: 50 µm in (**a**–**d**) and 10 µm in the inset in (**a**).

First of all, an evident morphological difference between the two populations was detected. LC cells were highly enlarged and showed larger nuclei than PC and the increase in nuclei dimension in LC was 57.85 23.23% (*p*-value < 0.05, see bars in Figure 2). Moreover, multinucleated cells were evident in LC (asterisks in Figure 2b), indicating cell senescence. By immunofluorescence, claudin 2 was distributed differently in the two populations of co-cultures. In PC, diffuse and non-homogeneous immunoreactivity was detected (Figure 2a) and some cell clusters did not even show claudin 2 positivity (Figure 2a, inset). Conversely, in LC, a clear staining was distributed along all cytoplasmic membranes (Figure 2b). The quantitative protein analysis by Western blot confirmed that claudin 2 expression increased in the LC vs. PC family, although this difference was not statistically significant (Figure 2e). Occludin immunolocalization was restricted to the cell membrane in the two considered populations but with a different pattern. In PC, continuous linear staining was evident (Figure 2c), while in LC, a punctate distribution was detected (Figure 2d). Once again, WB analysis was in accordance with the morphological data, and no differences in occludin expression were reported between PC and LC co-cultures (Figure S1).

**Figure 3:**
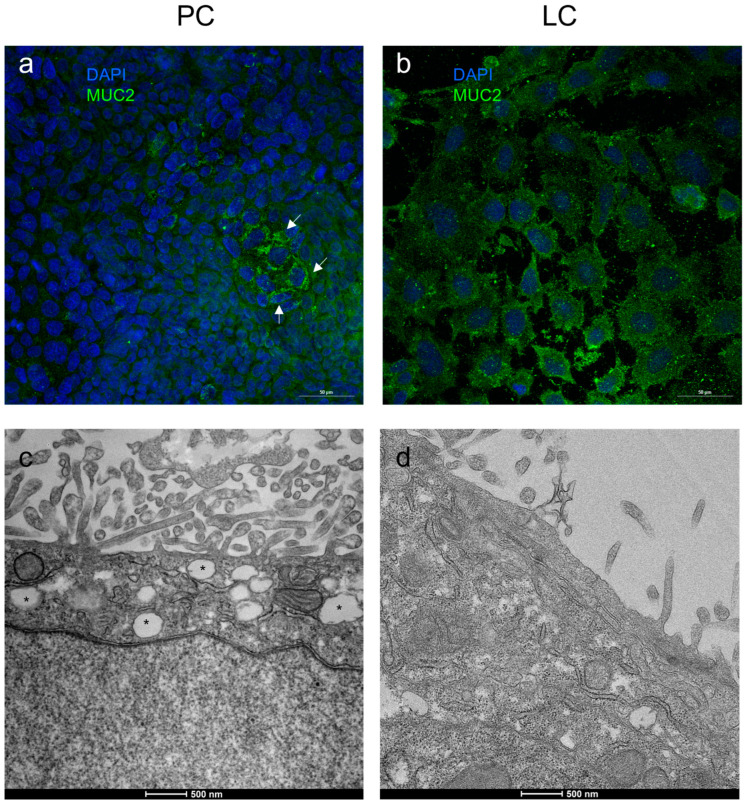
Mucus detection in Caco2/HT-29 70/30 co-culture showing different TEER values. (**a**,**b**): indirect immunofluorescence for MUC-2; (**c**,**d**): transmission electron analysis. PC: physiological co-cultures with TEER > 50 Ωcm^2^, in (**a**) Caco2 30th passage and HT-29 21st passage and in (**c**) Caco2 34th passage and HT-29 12th passage; LC: leaky co-cultures with TEER < 50 Ωcm^2^, in (**b**) Caco2 54th passage and HT-29 41st passage and in (**d**) Caco2 47th passage and HT-29 29th passage. Nuclei are counterstained with DAPI. MUC-2: mucin 2. White arrows in (**a**) and asterisks in (**c**) indicate the presence of granular mucus storage in PC. Bars: 50 µm in (**a**,**b**); 500 nm in (**c**,**d**).

As expected, MUC-2 was exclusively localized in the cytoplasm of co-cultures (Figure 3). In PC, MUC-2 granular immunoreactivity was always present (Figure 3), with several cell clusters exhibiting intense staining (Figure 3a, arrows). In LC, only scattered cells showed clear MUC-2 immunolocalization, which was fainter than in PC (Figure 3b). It is not possible to identify the goblet cells in the co-culture staining since, as previously reported in Caco2/HT-29 co-cultures, each cell line modifies the phenotype of the respective parental cells, partially acquiring unique morpho-functional properties [19]. By ultrastructural analysis, we confirmed an abundant mucus presence in PC (Figure 3c, asterisks) but not in LC (Figure 3d).

## 4. Discussion

We report for the first time that co-culture formed by the parental Caco2 and HT-29 cells, sub-cultivated for more than 40 and 20 passages, respectively, spontaneously present the typical features of an old leaky gut, i.e., cell dimension, IEB morphology, and functionality, allowing the possibility to explore the mechanisms bound to aging and IEB modification in the single parental cells (absorptive enterocytes, mucus-producing cells) and their co-culture.

**Figure 4:**
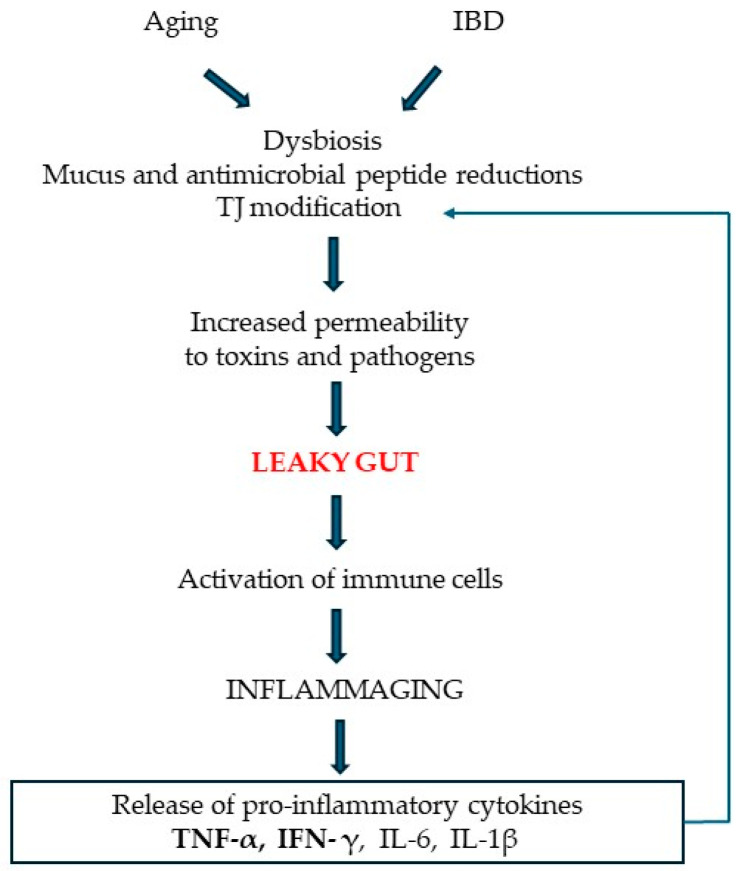
The sequence of events characterizing intestinal epithelial barrier damage during aging and intestinal bowel disease (IBD).

The most-used, more permeant intestine models are based on modified permeability, i.e., a leaky gut, by administering pro-inflammatory cytokines, such as TNF-α, IFN-γ, and/or LPS, to intestinal cells [29]. These models represent a leaky gut resulting from a pathological condition, such as a low grade of chronic inflammation (Figure 4).

Our model, in contrast, represents a leaky gut preceding a pathological situation, due to the physiological senescence of the intestinal cells. To the best of our knowledge, an in vitro model of age-associated intestinal epithelial barrier dysfunction was only obtained by treating Caco2 cells with D-galactose [30]. In vivo, a model of old mice and baboons showing higher leaky gut and associated dysfunctions was treated with a human-origin probiotic cocktail to ameliorate the problems [31]. The real novelty of our methodological approach is that, without any additional exogenous stimulus, the model allows the study of IEB impairment.

A possible explanation of these morpho-functional changes in co-culture with days in a culture of parental cells can be due to the high degree of heterogeneity in Caco2 morphology depending on the post-confluent days and the number of passages, all variables fully described both within the same in vitro culture and among cell cultures from different laboratories [32]. This heterogeneity profoundly affects cell function and limits the use of this in vitro intestinal cell model if not accurately considered. Since in our co-culture, Caco2 cells represent the dominant cytotype, the morpho-functional properties could be affected over time by increasing the number of their sub-cultivation passages. Indeed, we observed that in co-culture at high passage numbers, i.e., LC, nuclei were significantly larger than in PC, and multinucleated cells were present. Both these morphological features were already described for senescent cells [33] and are strongly suggestive of a process of physiological aging occurring in these cells over time.

The TEER values, together with the associated IEB permeability, are the main functional targets affected by Caco2 cell heterogeneity, thus representing key factors to consider when transport and absorption studies are performed, since they affect the bioavailability of drugs and supplements, for instance. TEER values can be influenced by different experimental conditions such as the days in culture, the growth medium, and the seeding density [34]. In our experimental conditions, the number of passages of the parental cell lines represents the only variable parameter able to affect the TEER of the co-cultures.

TEER and permeability are both governed by TJs even if in different ways. TEER represents a valuable tool for measuring the ionic conductance of the paracellular transport in the IEB, i.e., the flux of ions and solutes crossing the IEB through the TJs. In physiological conditions, the permeability of TJs is strictly controlled to avoid the passage of dangerous molecules. Still, many conditions can affect it, leading to a more open passage of molecules, including the dangerous ones. High TEER values indicate a high resistance to the passage of molecules, and they are associated with a low permeability; conversely, low TEER values are associated with a high permeability. The TEER values for physiological IEB vary among species and intestine segments. As it concerns ex vivo studies of humans, the Ussing chamber technique showed physiological values of the small intestine ranging from 50 to 100 Ωcm^2^, the same range of values detected in our in vitro PC model [17]. A value of TEER below 50 Ωcm^2^ is associated with lower resistance, thus indicating increased permeability and/or a leaky gut, such as in the case of the LC model, a situation indicating a perturbed IEB TJs structure, as evidenced in our experiments. The presence of a leaky gut has been associated with the onset of some pathologies such as coeliac disease, colorectal cancer, inflammatory bowel disease, diabetes, and degenerative disorders of the central nervous system [35].

**Figure 5:**
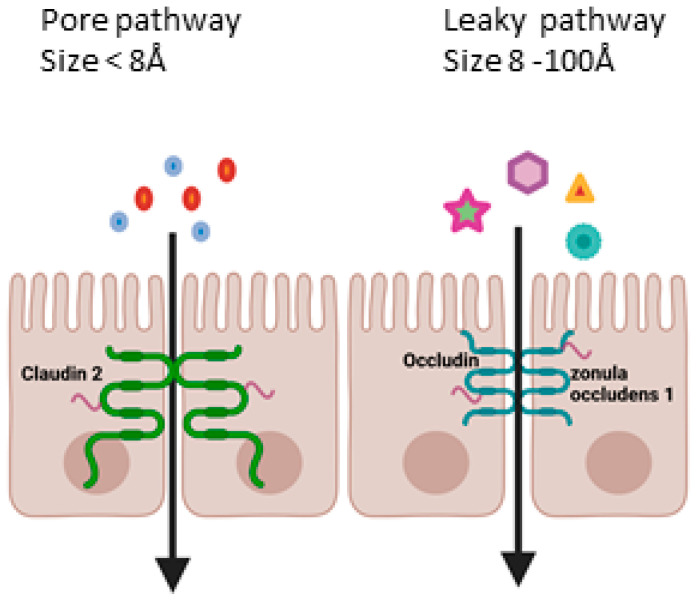
Simplified chart of paracellular permeability pathways (modified from [21] and created in BioRender. https://BioRender.com/n88k097 accessed on 26 February 2025).

Permeability depends on two different pathways: the pore pathway, which allows the passage of molecules depending on their size and charge, and the leaky pathway, allowing the passage of large ions and molecules, independently of charge (Figure 5). The pore pathway is mainly governed by claudins, while the leaky pathway is controlled by occludin and zonula occludens protein families [21].

The variation in TEER can be due to both different permeability pathways; therefore, the comprehension of which mechanism is involved requires the morphological identification of the TJ proteins actually involved. Integrating our previous and present results, we demonstrate that TEER decrease and permeability enhancement in LC is due to the modification of the pore formation since we observed a switch from the expression of the sealing protein claudin 1 to the permeabilizing protein claudin 2 herein reported. Finally, occludin expression was unchanged, as was ZO-1 expression [20].

The upregulation of claudin 2 in our experimental conditions agrees not only with the increased permeability observed in the co-culture model but also with other previously reported observations in physiological aging and human leaky gut. In the study by Man et al., mRNA expression of claudin 2 was significantly increased in terminal ileum biopsies of aging healthy subjects compared to younger ones [15]. In parallel, claudin 2 has been recognized as a key mediator of the leaky gut barrier in IBD [29]. More recently, a clinical study proposed claudin 2, coupled with neutrophil infiltration in the epithelium in IBD patients, as a valuable marker to predict clinical outcomes [36].

Many recent studies investigated occludin distribution in different experimental models involving Caco2 cells [11,13,37]. Data have demonstrated its main role in TJ formation, but in occludin-knock-out mice, TJ permeability was unaffected [38]. Other studies have demonstrated a delocalization of occludin involved in higher permeability of TJs [39], and our data are in accordance with a change in the localization pattern in LC co-cultures.

The presence of differentiated HT-29 cells as previously described [24] in our co-culture allowed us to also consider the importance and the modification of mucus by increasing the number of their sub-cultivations. The mucus layer does not affect directly TEER and permeability but physically contributes to the IEB activity by protecting it from host–microbial products. This mucus layer is produced by goblet cells, represented in our co-culture by HT-29 cells pre-differentiated as previously described [24]. The decrease in MUC-2 immunoreactivity observed in LC co-culture agrees with the reduction in goblet cells, and consequently of mucus production, observed in aging [40]. As IEB modifications are at the basis of a weak, but chronic, inflammatory state, we investigated the possibility that LC exhibiting molecular modifications in TJ composition, mimicking leaky/aged gut, could be more susceptible to an inflammatory milieu. Pro-inflammatory cytokines TNF-α and INF-γ are known to induce IEB modifications through the rearrangement of TJ proteins as determined in animal and in vitro studies [41,42,43]. As regards the mechanisms involved in the modulation of TJ by cytokines, the most relevant one appears to be the activation of myosin light chain kinase (MLCK), the point of convergence of the signaling pathways triggered by TNF-α and INF-γ. The phosphorylation of the myosin light chain by MLCK regulates several cytoskeleton proteins leading to the modification of the tight junction scaffold and the redistribution of occludin [42]. Other events have been described, such as the TNF-α-induced activation of ERK 1/2 and the downstream transcription factors activator protein 1 (AP-1) and Elk-1, which can increase claudin 2 expression [44,45] and the IFNγ-induced AMPK activation, which can reduce occludin and ZO-1 expression [43].

Both TNF-α and INF-γ are described to induce increases in paracellular permeability by affecting to a greater extent the leaky pathway rather than the pore pathway. Therefore, their administration to LC co-cultures leads to a higher reduction in IEB integrity, i.e., TEER, compared to PC, associated with an increase in permeability.

The morpho-functional modifications described here for LC, together with the setting up of the co-culture model through the spontaneous aging of parental cells, agree with the results obtained in human studies [15,36] and offer the possibility to clarify at a cellular and molecular level the still-unknown processes involved.

## 5. Conclusions

Considering the present results, we can consider the LC co-culture as an appropriate in vitro model of gut aging obtained exclusively by increasing the passage number of parental cells, characterized by a significant reduction in TEER, the absence of significant variation in occludin, and the increased level of claudin 2. The present study agrees with the observation that tight junction remodeling can spontaneously occur in in vitro intestinal cell cultures [46] and that aging is associated both in vitro and in vivo with increased IEB permeability.

Future studies will consider the onset of inflammation consequences by implementing the model of in vitro Caco2/HT-29 70/30 co-culture, adding cell populations present in the gut lamina propria in patients affected by intestinal bowel diseases.

## Figures and Tables

**Table 1:** Antibodies and protocols for indirect immunofluorescence analysis. CLDN2: claudin 2; MUC-2: mucin 2. RT: room temperature; NGS: Normal Goat Serum; PBS: Phosphate-Buffered Saline; BSA: Bovine Serum Albumin.

	CLDN2	OCCLUDIN	MUC-2
Block of nonspecific binding sites	30’ at RT in NGS 1:10 in PBS 0.1 M pH 7.4	30’ at RT in NGS 1:10 in PBS 0.1 M pH 7.4	30’ at RT in NGS 1:10 and BSA 5% in PBS 0.1 M pH 7.4
Primary antibody	Mouse anti-human claudin 2 (Invitrogen, Monza (MB), Italy, #32-5600) 1:100 in PBS 0.1 M pH 7.4 for 4 h at RT	Rabbit anti-human occludin (Invitrogen, Monza (MB), Italy, #MA3-012) 1:100 in PBS 0.1 M pH 7.4 overnight at 4 °C	Mouse anti-human MUC-2 Merck Italy (Milan, Italy, #1412598) 1:10 in 3% BSA/PBS 0.1 M pH 7.4 for 1 h at 37 °C
Secondary antibody	Alexa Fluor 488 goat anti-mouse (ThermoFisher Scientific, Waltham, MA, USA) dilution 1:200 in PBS 0.1 M pH 7.4, 1 h at RT in the dark	Alexa Fluor 488 goat anti-rabbit (ThermoFisher Scientific, Waltham, MA, USA) dilution 1:200 in PBS 0.1 M pH 7.4, 1 h at RT in the dark	Alexa Fluor 488 goat anti-mouse (ThermoFisher Scientific, Waltham, MA, USA) dilution 1:200 in PBS 0.1 M pH 7.4, 1 h at RT in the dark

## Data Availability

Dataset available on request from the authors.

## Supplementary Materials

The following supporting information can be downloaded at: https://www.mdpi.com/article/10.3390/biology14030267/s1, Figure S1: Densitometric quantification of western blot analysis for occludin. The values were normalized to β-actin and expressed as a fraction of PC normalized to 1. Mean values ± SEM (n = 3). PC: physiological co-cultures with TEER > 50 Ωcm^2^, Caco2 30th passage and HT-29 21st passage; LC: leaky co-cultures with TEER < 50 Ωcm^2^, Caco2 54th passage and HT-29 41st passage. OCLN: occludin.

## Abbreviations

The following abbreviations are used in this manuscript:

BSA	Bovine Serum Albumin
CLN2	Claudin 2
IBD	Inflammatory Bowel Disease
IBS	Irritable Bowel Syndrome
IEB	Intestinal Epithelial Barrier
IFN-γ	Interferon-γ
LC	Leaky co-cultures with TEER values < 50 Ωcm^2^
LPS	Lipopolysaccharide
LY	Lucifer Yellow
MLCK	Myosin Light Chain Kinase
MUC-2	Mucin 2
NGS	Normal Goat Serum
PBS	Phosphate-Buffered Saline
PC	Physiological co-cultures with TEER values > 50 Ωcm^2^
ROS	Reactive Oxygen Species
TEER	Transepithelial Electrical Resistance
TEM	Transmission Electron Microscopy
TJs	Tight Junctions
TNF-α	Tumor Necrosis Factor-α
ZO	Zonula Occluden
