# Supplementary figures and images for: Caco2/HT-29 In Vitro Cell Co-Culture: Barrier Integrity, Permeability, and Tight Junctions’ Composition During Progressive Passages of Parental Cells
Source: Biology (Basel). 2025 Mar 6;14(3):267. doi: 10.3390/biology14030267 (PMC11939685; doi:10.3390/biology14030267)

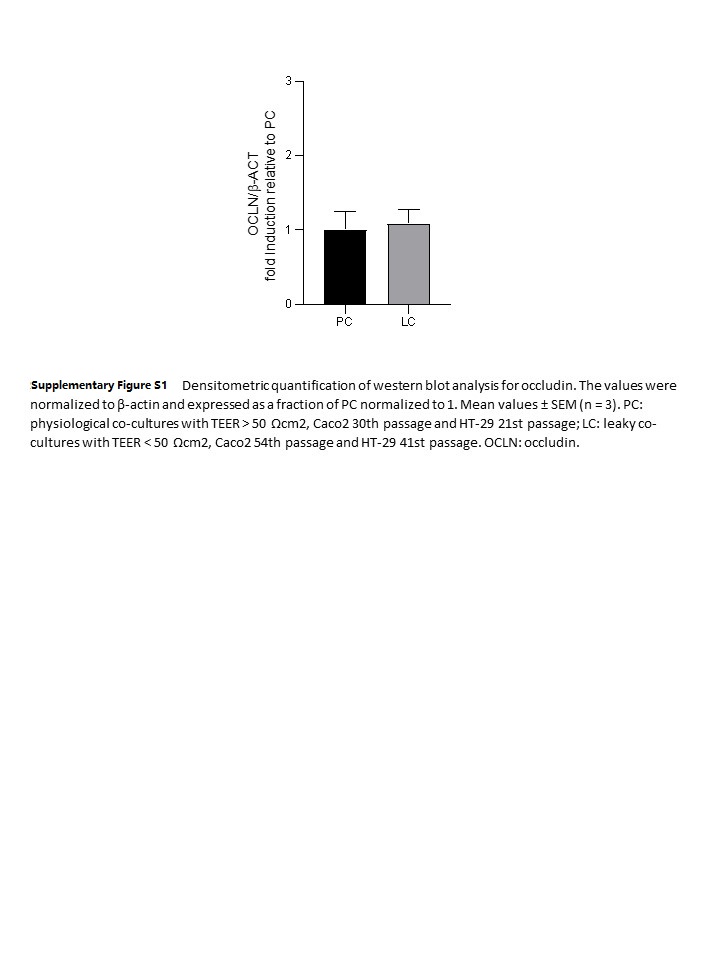

Supplement: Supplementary file 1 [file biology-14-00267-s001.zip › biology-3488204-supplementary.jpg]
